# Modulation of the Human Erythrocyte Antioxidant System by the 5- and 6-Membered Heterocycle-Based Nitroxides

**DOI:** 10.3390/molecules29122941

**Published:** 2024-06-20

**Authors:** Krzysztof Gwozdzinski, Stella Bujak-Pietrek, Anna Pieniazek, Lukasz Gwozdzinski

**Affiliations:** 1 Department of Oncobiology and Epigenetics, Faculty of Biology and Environmental Protection, University of Lodz, 90-236 Lodz, Poland; krzysztof.gwozdzinski@biol.uni.lodz.pl (K.G.); anna.pieniazek@biol.uni.lodz.pl (A.P.); 2 Department of Chemical Hazards, Nofer Institute of Occupational Medicine, 91-348 Lodz, Poland; stella.bujak@imp.lodz.pl; 3 Department of Pharmacology and Toxicology, Medical University of Lodz, ul. Zeligowskiego 7/9, 90-752 Lodz, Poland

**Keywords:** nitroxide, red blood cells, oxidative stress, lactate dehydrogenase, superoxide dismutase, catalase

## Abstract

Nitroxides are stable radicals consisting of a nitroxyl group, >N-O^•^, which carries an unpaired electron. This group is responsible for the paramagnetic and antioxidant properties of these compounds. A recent study evaluated the effects of pyrrolidine and pyrroline derivatives of nitroxides on the antioxidant system of human red blood cells (RBCs). It showed that nitroxides caused an increase in the activity of superoxide dismutase (SOD) and the level of methemoglobin (MetHb) in cells (in pyrroline derivatives) but had no effect on the activity of catalase and lactate dehydrogenase. Nitroxides also reduced the concentration of ascorbic acid (AA) in cells but did not cause any oxidation of proteins or lipids. Interestingly, nitroxides initiated an increase in thiols in the plasma membranes and hemolysate. However, the study also revealed that nitroxides may have pro-oxidant properties. The drop in the AA concentration and the increase in the MetHb level and in SOD activity may indicate the pro-oxidant properties of nitroxides in red blood cells.

## 1. Introduction

Nitroxides are stable organic radicals having a nitroxyl group, >N-O^•^, with an unpaired electron, which determines their properties [1]. The presence of the nitroxyl group means that these radicals can undergo oxidation or reduction reactions to diamagnetic compounds, i.e., oxoammonium cations and hydroxylamines. The most famous nitroxides include 2,2,6,6-tetramethylpiperidine derivatives, although 2,2,5,5-tetramethylpyrroline, 2,2,5,5-tetramethylpyrrolidine, and 1,3-oxazolidine derivatives are also used. In addition to their applications as spin labels and spin probes in electron paramagnetic resonance spectroscopy in biology and medicine, nitroxides have also become known as antioxidants [2,3].

The first nitroxides were synthesized by Rozantsev’s group in the early 1960s. their synthesis methods and properties were presented in the book Rozantsev wrote [4]. In turn, the properties of nitroxides and their use as probes and spin labels in labeling biological molecules were widely described in works edited by Berliner [5]. Another book on nitroxide chemistry published many years later was Kocherginsky and Swartz [6]. Interesting information about nitroxides can also be found in the review [7].

The properties of nitroxides depend on the structure of the heterocyclic ring, the type of substituent, the electric charge, and their hydrophilic or lipophilic nature. For example, piperidine nitroxides, which have no electrical charge, are reduced faster in cells than pyrroline and pyrrolidine nitroxides [8,9]. The nature and size of substituents in the heterocyclic ring may affect the permeation of nitroxides across the plasma membrane [10]. The charge introduced into the nitroxide molecule affects the rate of penetration through cell membranes and, consequently, the rate of their reduction inside the cells [9].

Nitroxides engage as radicals in recombination reactions with hydroxyl radical (HO^•^, alkyl R^•^) and peroxide (ROO^•^) but not oxygen-centered radicals (alkoxyl, RO^•^) [11,12,13,14]. Recently, it was shown on the example of piperidine nitroxides that the rate constants of interaction with peroxide radicals depend on the type of substituent in the 4-position-5.1 × 10^6^ M^−1^ s^−1^ Tempo as 1.1 × 10^6^ M^−1^ s^−1^ for Tempol and Tempamine 5.4 × 10^5^ M^−1^ s^−1^ to the Tempone 5.6 × 10^4^ M^−1^ s^−1^ [15]. Nitroxides oxidize transition-metal ions in lower oxidation states to higher oxidation states, preventing Fenton reactions [16,17]. They also protect cells and tissues against the oxidizing and nitrating properties of peroxynitrite (ONOO^−^), which is formed in the reaction of superoxide with nitric oxide in vivo and in vitro. Nitroxide does not react directly with ONOO^−^ but rather reacts with the oxoammonium cation. The reaction product is reconstituted nitroxide, which proves the catalytic effect of nitroxides [18]. The reaction rate constant ranges from 6 × 10^6^ M^−1^ s^−1^ for Tempo to 2.7 × 10^6^ M^−1^ s^−1^ for 3-carbamoyl-2,2,5,5-tetramethylpyrrolidine-1-oxyl. Moreover, they also have pseudo-dismutase activity in the dismutation of superoxide anion (O_2_^•−^) [19]. Nitroxides also induce the pseudo-catalase activity of heme proteins in the decomposition of hydrogen peroxide (H_2_O_2_) and also reduce ferryl forms of myoglobin (MbFe(IV) = O) or radical-form (MbFe(IV) = O^•^) derivatives [20]. Similar ferryl forms are also formed in the case of hemoglobin [21]. Nitroxides alleviate the effects induced by H_2_O_2_ in spontaneously immortalized fibroblasts (B14) but do not protect against the lipid peroxidation induced by H_2_O_2_. On the other hand, they inhibit the lipid peroxidation induced by doxorubicin and have a cytoprotective effect, increasing their survival [22]. Nitroxides also have pharmacological effects as potential anticancer drugs, but they also inhibit the harmful effects of anticancer drugs such as doxorubicin, paclitaxel, and docetaxel in vitro and in vivo [23,24,25]. Moreover, they inhibit the phenomenon of ferroptosis [26,27], reduce inflammation caused by *Mycobacterium tuberculosis* [28], and also protect against retinopathy [29], as well as ischemia and reperfusion [30].

Nitroxides are effective radioprotectors used as a contrast agent in MRI [31,32,33]. It was shown that sublytic concentrations of hydrogen peroxide, together with the nitric oxide (NO^•^) donor SNAP (*S*-nitroso-*N*-acetyl-d, l-penicillamine), lead to oxidative/nitroso stress through activation of the p38 MAPK and p53 cascades, as well as DNA damage and tyrosine nitration in proteins. Of the six antioxidants, including SOD, CAT, Tempo, *N*-acetylcysteine, dimethylthiourea, and uric acid, Tempo is the best antioxidant for reducing the changes initiated by H_2_O_2_ and SNAP, including the activation of the p38 MAPK and p53 stress cascades and the production of ROS, NO^•^, and peroxynitrite, as well as double-strand DNA breaks and tyrosine nitration in proteins [34].

Superoxide dismutase (SOD) is an important antioxidant protein found in RBCs that catalyzes the dismutation of O_2_^•−^ to hydrogen peroxide. H_2_O_2_ is then broken down into oxygen and water by catalase (CAT) or glutathione peroxidase (GPx). All these enzymes perform an important role in protecting RBCs against oxidative stress. Due to its function, the red blood cell is constantly exposed to high concentrations of oxygen and its toxic metabolites [35,36]. In addition, the enzymatic system is supported by low-molecular-weight antioxidants, such as glutathione, ascorbic acid, tocopherols, and others, which inactivate reactive oxygen species [35]. Lactate dehydrogenase (LDH) is an enzyme that catalyzes the reversible conversion of lactate to pyruvate with the reduction of NAD^+^ to NADH and is an important oxidoreductase of the anaerobic carbohydrate metabolic pathway [37]. Relatively recently, it was shown that superoxide can induce and enhance the production of hydrogen peroxide in the aqueous phase, which is catalyzed by LDH. However, the physiological significance of this finding is unknown [38].

**Figure 1:**
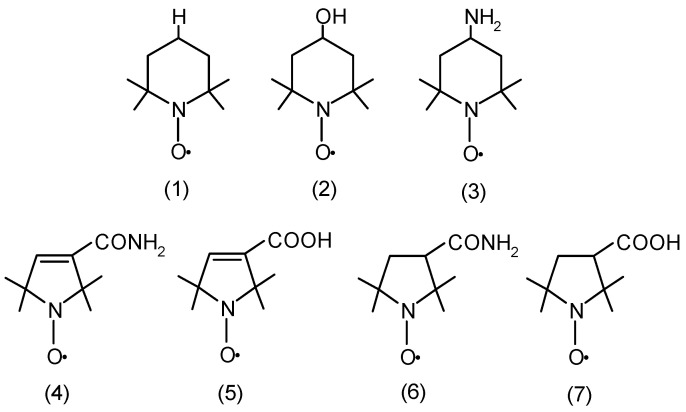
Chemical structures of piperidine (Tempo (**1**), Tempol (**2**), and Tempamine (**3**)), pyrroline (Pyrrolin (**4**) and Carboxy-Pyrrolin (**5**)); and pyrrolidine (Pyrrolid (**6**) and Carboxy-Pyrrolid (**7**)) nitroxides.

Recently, the properties of nitroxides and their effects have been discussed in experiments conducted on animals, as well as in clinical trials in various pathological conditions accompanied by oxidative stress [39]. In light of the current research, nitroxides can protect cells and tissues against reactive oxygen species. They were also used as antioxidants in the in vivo treatment of breast cancer in rats [25,40]. Our previous studies related to the participation of GSH in the reduction of nitroxides and glutathione-dependent enzymes prompted us to determine the level of thiol groups in both membrane proteins and hemolysate after the treatment of RBCs with nitroxides [41]. Glutathione and thiols perform an important role in the redox systems. This especially applies to peptides and low-molecular-weight proteins. Because they are very sensitive to oxidation, they are excellent antioxidants for protecting cells and tissues against oxidative stress [42,43]. However, the mechanism of their interactions with enzymatic proteins and low-molecular-weight cellular antioxidants, such as glutathione and ascorbate, is not fully understood. Therefore, in this study, an attempt was made to determine the effects of nitroxide radicals on the activity of antioxidant enzymes, including superoxide dismutase (SOD) and catalase (CAT), as well as LDH and methemoglobin (MetHb), in human RBCs. The concentration of ascorbic acid in the RBCs and the levels of thiols in the membranes and hemolysate were determined. Oxidative stress indicators were also determined, such as the level of substances that are reactive with thiobarbituric acid and the level of carbonyl compounds. In our studies, we used four substances based on 5-membered heterocycle nitroxides: the pyrroline derivatives 3-carbamoyl-2,2,5,5-tetramethyl-3-pyrroline-1-oxyl (**4**) (Pyrrolin) and 3-carboxy-2,2,5,5-tetramethyl-3-pyrroline-1-oxyl (**5**) (Carboxy-Pyrrolin) and the pyrrolidine derivatives 3-carbamoyl-2,2,5,5-tetramethyl-3-pyrrolidine-1-oxyl (**6**) (Pyrrolid) and 3-carboxy-2,2,5,5-tetramethyl-3-pyrrolidine-1-oxyl (**7**) (Carboxy-Pyrrolid) (Figure 1). The research used RBCs, the most numerous of the morphotic blood components, which are characterized by a relatively simple structure compared to other cells.

## 2. Results

**Figure 2:**
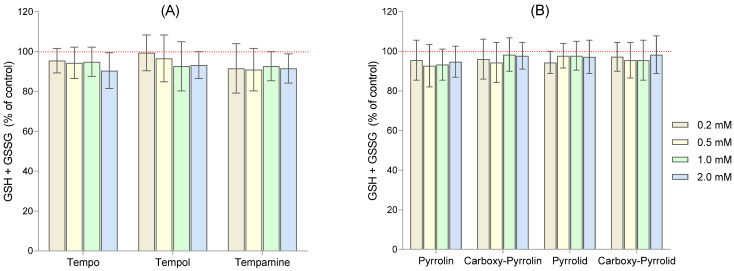
Values of total glutathione concentration (GSH + GSSG) in RBCs after treatment with various concentrations of nitroxides, including (**A**) six-membered nitroxides, and (**B**) five-membered nitroxides. The results are presented as percentage changes compared to the control, the value of which was taken as 100% (n = 9).

Since six- and five-membered nitroxides in RBCs led to a strong decrease in the intracellular GSH concentration, we determined the level of total glutathione (GSH + GSSG) after their incubation with the seven nitroxides (Figure 2A,B).

**Figure 3:**
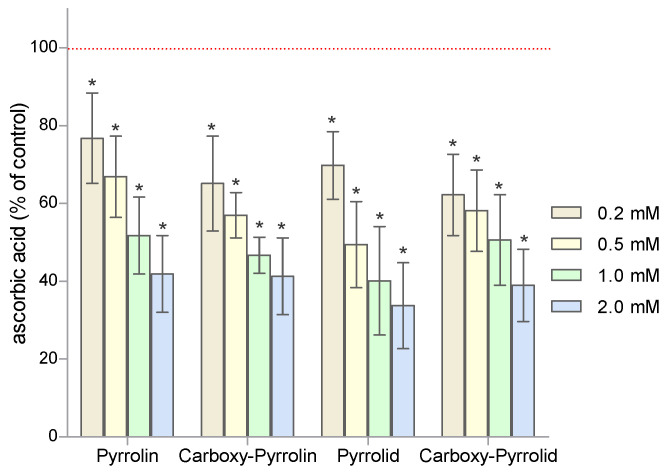
The levels of ascorbic acid in RBCs after treatment with five-membered nitroxide radicals. The results are presented as percentage changes compared to the control, the value of which was taken as 100% (n = 8). (* significantly different in comparison to the control at least at *p* < 0.05).

The effect of nitroxides on the level of ascorbate in human RBCs was investigated using bathophenanthroline. The obtained results clearly showed that the concentration of ascorbic acid in RBCs incubated with nitroxides decreased significantly with the increasing nitroxide concentration (Figure 3). Pyrrolidine nitroxides caused a slightly greater decrease in ascorbate than pyrroline derivatives. These results confirm the fact that ascorbate performs an important role in the reduction of nitroxides in RBCs. The results are presented as percentage changes relative to the control.

**Figure 4:**
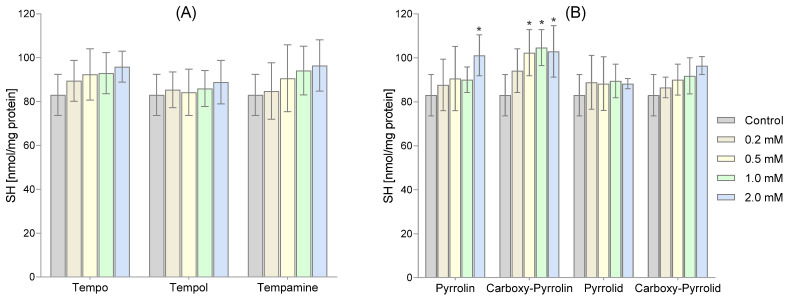
The thiol group concentrations in membrane proteins at various concentrations of tested piperidine (**A**), pyrroline, and pyrrolidine (**B**) nitroxides. The obtained results are presented as mean with standard deviation (n = 8). (* significantly different in comparison to the control at least *p* < 0.05).

We measured the amount of thiol groups present in both membrane and hemolysate proteins. Our findings indicated that the six-membered and five-membered nitroxides caused an increase in the -SH group content, as shown in Figure 4A,B. However, only pyrroline nitroxide significantly increased the SH group content. We observed this increase in the highest concentration of pyrroline nitroxide and the three highest concentrations of Carboxy-Pyrollin, as depicted in Figure 4B.

**Figure 5:**
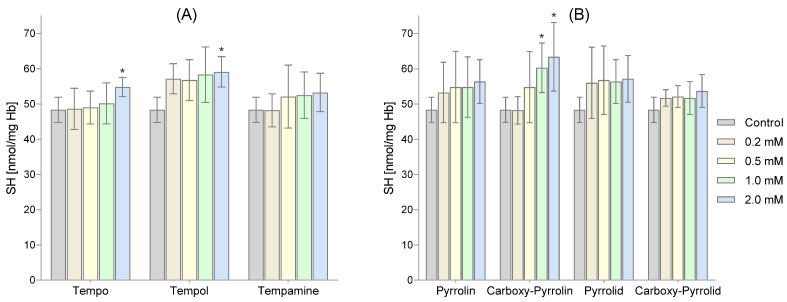
The thiol group concentration in hemolysate proteins at various concentrations of tested piperidine (**A**), pyrroline, and pyrrolidine (**B**) nitroxides. Results shown include mean ± SD values (n = 8). (* significantly different in comparison to the control at least *p* < 0.05).

Figure 5 shows the level of thiol groups in hemolysate proteins. Among the six-membered nitroxides, only the highest concentrations of Tempo and Tempol caused a significant increase in the content of -SH groups in membrane proteins (Figure 5A). However, in the case of five-membered nitroxides, only Carboxy-Pyrrolin used in the two highest concentrations increased the level of thiol groups (Figure 5B).

**Figure 6:**
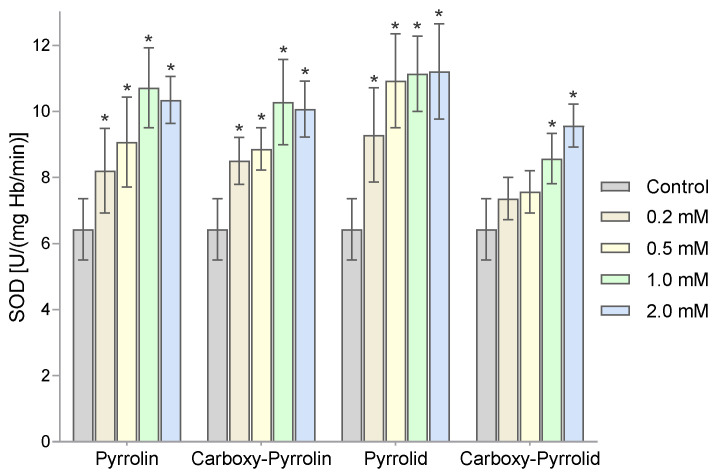
The activity of superoxide dismutase in human RBCs treated with different concentrations of nitroxides. The results presented are mean ± SD values (n = 10). (* significantly different in comparison to the control at least *p* < 0.05).

Then, the activity of superoxide dismutase was assessed using the adrenaline method. Figure 6 shows the changes in the activity of superoxide dismutase in human RBCs after 1 h of incubation with increasing concentrations of nitroxides. A significant increase in SOD activity already occurred at concentrations of 0.2 mM of the nitroxides Pyrrolin, Carboxy-Pyrrolin, and Pyrrolid. However, for Carboxy-Pyrrolid, it only occurred at a concentration of 1 mM. The highest increases in the activity of this enzyme were observed for the highest concentrations of Pyrrolid and the lowest of Carboxy-Pyrrolid.

**Figure 7:**
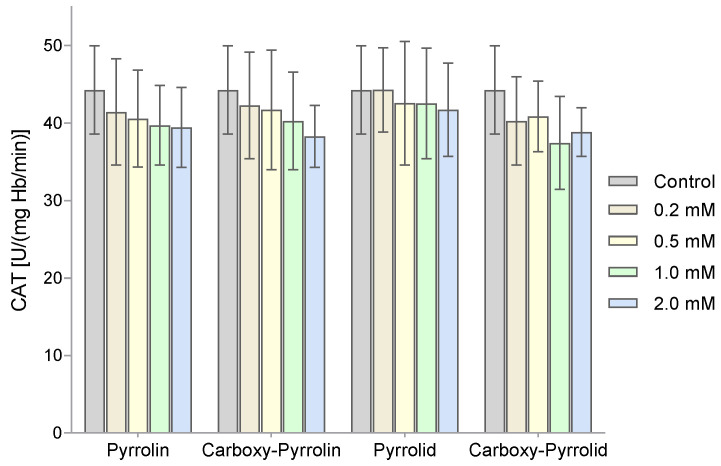
Catalase activity in RBC lysates after their previous treatment with various concentrations of penta-fronted nitroxides. The results presented are mean ± SD values (n = 10).

The next enzyme tested was catalase. A decrease in the activity of this enzyme was observed in Figure 7, but no statistically significant differences were found in the catalase activity of RBCs treated with different concentrations of nitroxides.

**Figure 8:**
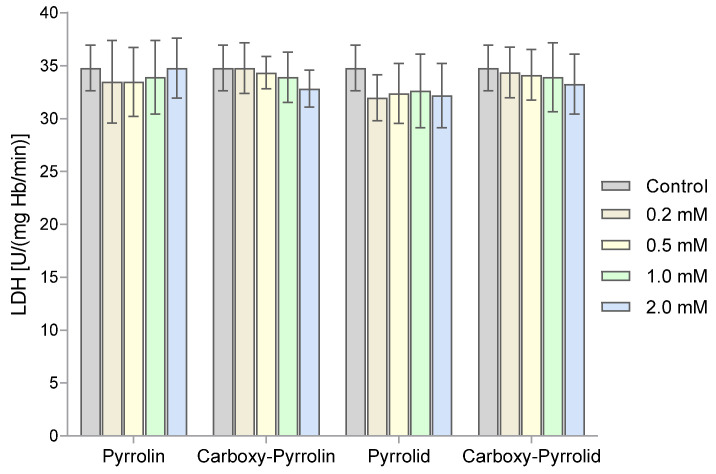
Lactate dehydrogenase activity determined in RBCs after their treatment with pentameric nitroxides. The results presented are mean ± SD values (n = 7).

The effect of nitroxides on the activity of lactate dehydrogenase in RBCs was also examined. However, the results obtained indicate that the nitroxides used did not affect the activity of this enzyme (Figure 8).

**Figure 9:**
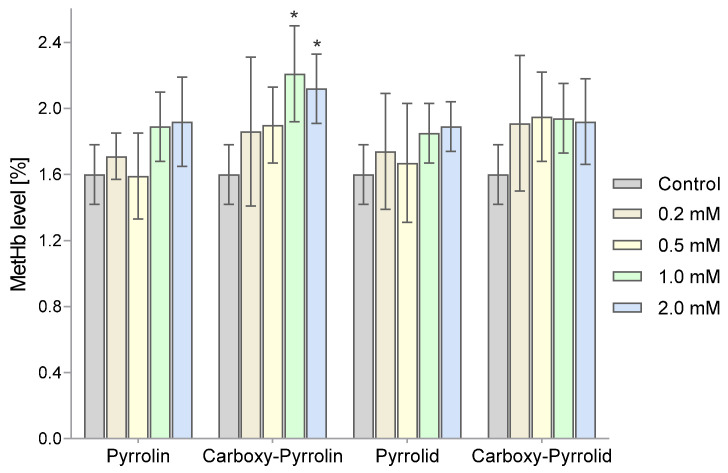
Changes in the content of MetHb relative to the total hemoglobin pool after treatment of RBCs with nitroxides. The results presented are mean MetHb ± SD of concentrations (n = 8). (* significantly different in comparison to the control at least at *p* < 0.05).

The percentage of methemoglobin in the total hemoglobin pool was also determined. Figure 9 shows changes in the MetHb content relative to total hemoglobin after an hour of incubation of the RBC suspensions with the tested nitroxides. A statistically significant increase in MetHb levels was observed in the blood cells exposed to the highest concentrations (1 mM and 2 mM) of pyrroline nitroxides (Pyrrolin and Carboxy-Pyrrolin).

**Figure 10:**
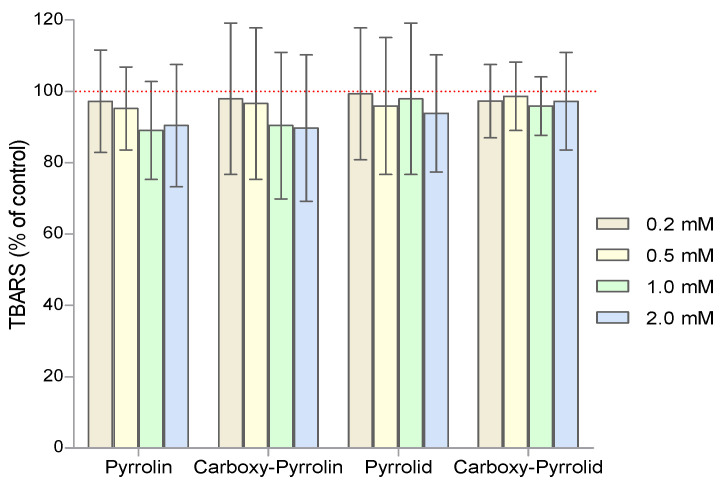
Assessment of the degree of lipid peroxidation in RBCs after treatment with increasing concentrations of nitroxides. The obtained results (mean values ± SD) were calculated with the control, the value of which was taken as 100% (n = 8).

Since nitroxides also have pro-oxidant properties, the degree of lipid peroxidation with thiobarbituric acid was assessed. High (1 mM and 2 mM) concentrations of pyrroline nitroxides, as opposed to pyrrolidine nitroxides, showed a slight decrease in the degree of products reacting with TBA, but these results were not statistically significant (Figure 10).

**Figure 11:**
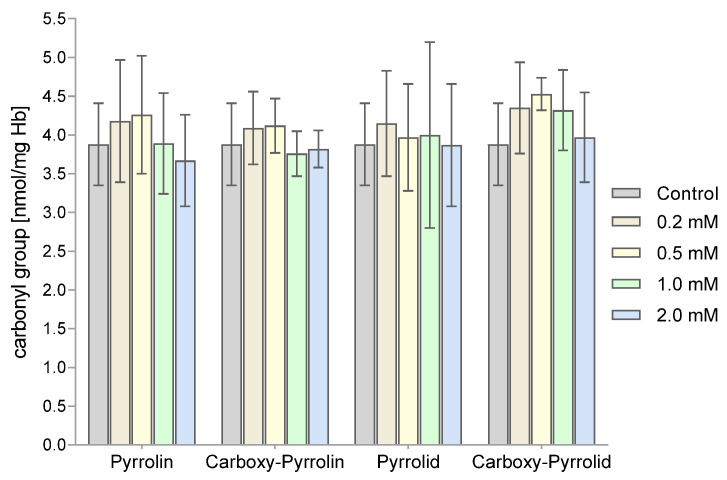
Assessment of the degree of oxidative damage to proteins (level of carbonyl groups) in RBCs after their treatment with nitroxides derived from pyrroline and pyrrolidine. The presented results are the average ± SD of 8 independent measurements.

The levels of carbonyl groups were also determined as a measure of the degree of oxidative damage to proteins. The levels of the carbonyl groups of RBC proteins did not change significantly after their incubation with the tested nitroxides (Figure 11).

## 3. Discussion

In our previous work [41], we examined the effects of three piperidine nitroxides, i.e., Tempo (**1**), Tempol (**2**), and Tempamine (**3**), on the antioxidant system in human red blood cells. These derivatives differed in the presence of different substituents in the four-position of the piperidine ring. In the current work, we tested nitroxyl derivatives of 2,2,5,5-tetramethopyrroline and 2,2,5,5-tetrametopyrrolidine, which contained the same substituents in the 3-position of the heterocyclic ring. Although the nitroxyl group plays a key role in redox reactions, the influence of the substituents and the structure of the heterocyclic ring are irrelevant. Piperidine nitroxides have a very different spatial structure compared to five-membered derivatives. Piperidine nitroxides have a chair structure, while the structure of pyrroline derivatives is flat due to the presence of a double bond in the ring. Only the nitroxyl group “protrudes” above the plane of the heterocyclic ring. However, in the case of pyrrolidine nitroxides, the structure of the heterocyclic ring is not flat, and the atoms constituting the ring do not lie in one plane but protrude above and below the plane. Interestingly, the half-life of piperidine nitroxides in red blood cells is approximately 1 h. For comparison, the half-life of five-membered nitroxides is at least five to six times longer.

In the cell, nitroxides are reduced to hydroxylamines by ascorbate and NADPH [44]. Nitroxides can also be reduced by thiols [45,46]. However, thiols also participate in the reduction of nitroxides in cells. We showed that inhibitors of thiol groups (such as Hg(II), p-chloromercuric benzoic acid, and *N*-ethylmaleimide) reduced the rate of nitroxide reduction in RBCs [9]. In our previous studies, we showed that nitroxides reduced the level of glutathione in red blood cells [47]. In the case of piperidine nitroxides (Tempo, Tempol, and Tempamine) and pyrrolidine nitroxides at the highest concentrations, the GSH concentration dropped below 10%, while for pyrroline nitroxides, it only dropped by 55–60%, which clearly shows the influence of the structure of the heterocyclic ring. The decrease in glutathione concentration caused by Tempo in RBCs was also observed by other authors [48]. We also found that nitroxides decrease the activity of glutathione-dependent enzymes such as glutathione peroxidase (GPx), glutathione transferase (GT), and glutathione reductase (GRx) in RBCs [49]. Since in previous work, we showed a decrease in GSH concentration and a decrease in the activity of GSH-dependent enzymes for five- and six-membered nitroxides, we now determined the levels of total glutathione (GSH + GSSG) in RBCs after treatment with piperidine, pyrroline, and pyrrolidine nitroxides. We did not find any changes in the total glutathione pool (Figure 2). These results indicate that glutathione did not form conjugates with nitroxides that could be transported outside cells. On the other hand, we showed that glutathione itself, unlike ascorbic acid, did not cause nitroxide reduction over 1 h even when its excess in relation to nitroxide was 100 times higher [10]. The reduction rate depended on the AA concentration and the presence of oxygen. In anaerobic conditions, the reduction rate was much higher than in aerobic conditions [10]. Moreover, we found that the rate of reduction of piperidine nitroxides depended on the substituent in the 4-position. For example, Tempol and Tempace (4-acetamido-2,2,6,6-tetramethylpiperidine-1-oxyl) were reduced faster by AA than Tempo, which is correlated with the electronegativity of functional groups. Interestingly, the decrease in glutathione concentration in RBCs was much greater in the case of piperidine and pyrrolidine nitroxides than in the case of pyrroline nitroxides [47]. Reduction of nitroxides in RBCs occurs mainly by reacting with ascorbic acid, not by directly reacting with glutathione. It appears that the decrease in glutathione concentration in RBCs is not related to its direct oxidation by nitroxide but to the regeneration of DHA (dehydroascorbate) to ascorbate by GSH [50]. DHA is a product that is produced in the reduction of nitroxides by ascorbic acid.

Figure 3 shows that the derivatives of pyrrolidine nitroxides reduced the concentration of ascorbic acid in RBCs. The most significant decrease in AA concentration in RBCs occurred after the treatment with Pyrrolid. In turn, in RBCs, the decreases in ascorbic acid concentration were greater in the cases of six-membered nitroxides than in the cases of five-membered nitroxides. The nitroxides Tempol and Tempamine, but also Pyrrolid (**6**), most significantly reduced the concentration of ascorbic acid in RBCs [47]. However, lower effectiveness in reducing AA concentration was observed for Tempo, Pyrrolin, Carboxy-Pyrrolin, and Carboxy-Pyrrolid. Comparing the midpoint potential (mV) versus the normal hydrogen electrode, slight differences in the normal potentials were found for Tempol, Tempamine, and Carboxy-Pyrrolid [3].

Although the treatment of RBCs with nitroxides led to a decrease in GSH concentrations, in the case of thiols, an increase in the level of thiol groups was observed, although it was statistically insignificant. Only in the cases of the highest concentrations of Pyrrolin and the three highest concentrations of Carboxy-Pyrrolin was the increase in -SH groups statistically significant. Similar observations related to the increase in -SH groups concerned hemolysate proteins. This time, a statistically significant increase was observed for the highest concentrations of piperidine nitroxides Tempo and Tempol and the two highest concentrations of Carboxy-Pyrrolin (approx. 30%). Since these results concern proteins present in membranes and hemolysate, it seems that they did not participate in the redox cycle with the nitroxides. On the other hand, these results indicate that nitroxides can stimulate an increase in thiol groups in RBCs. Red blood cells contain many enzymatic proteins rich in thiol groups, such as peroxyredoxin2 (Prx2), thioredoxin (Trx), thioredoxin reductase, and glutaredoxins (Grxs) in which -SH groups perform a catalytic role [51]. For example, Prx2 can react with H_2_O_2_ and peroxynitrite very quickly, with rate constants of 1 × 10^8^ and 1.4 × 10^7^ M^−1^ s^−1^, respectively [52,53]. Moreover, Prx2 prevented the oxidation of Hb and stabilized its structure. It has been shown that Prx2 knockout mice were exposed to increased Hb oxidation, Heinz body formation, and hemolytic anemia [54]. A relatively small part of Prx2 is located in the plasma membrane and is associated with the cytoplasmic part of the band 3 protein [55]. Thioredoxin (Trx) is a monomer with a low molecular weight (12 kDa) that is rich in cysteine residues, which ultimately reduce protein disulfide bridges. The intramolecular disulfide formed in Trx is reduced by thioredoxin reductase (TR1). The homodimeric thioredoxin reductase contained in RBCs is a flavoprotein containing selenocysteine in its active site, and each subunit contains FAD- and NADPH-binding domains. Thioredoxin reductase restores the active (reduced) forms of thioredoxin and peroxyredoxin2. Glutaredoxins (Grxs) are cysteine-dependent enzymes that catalyze the formation and reduction of mixed disulfides formed between the thiol groups of proteins and GSH [56]. Grx1 and Grx3 are present in RBCs. Grxs may contain one (monothiol Grx) or two (dithiol Grx) -SH group. Monothiol Grxs are not oxidoreductases and have only one cysteine residue in the active site, which is used together with GSH to form and transfer iron–sulfur ([Fe–S]) clusters to proteins [51]. Grx1 contains two cysteine residues and reduces protein disulfides and protein–GSH mixed disulfides (deglutathionylation) [57].

By examining the activity of superoxide dismutase, we showed an increase in the activity of this enzyme after the treatment of RBCs with all nitroxides. However, Pyrrolid at the highest concentration caused a greater increase in SOD activity (approx. 75%). A smaller increase in SOD activity was observed for both pyrroline nitroxides. In turn, the lowest increase in SOD activity was recorded in the case of Carboxy-Pyrrolid (approx. 42%) (Figure 5). The increase in SOD activity may indicate overproduction of superoxide anion in RBCs after treatment with nitroxides.

It was shown that common conventional active ingredients of pesticides, such as carbaryl, permethrin, lindane, and malathion, used in the home and garden sector initiated oxidative stress in various cells and tissues, as well as among experimental animals [58]. For example, malathion in many cells caused an increase in SOD activity, which was observed in rat RBCs and in small Swiss mice, among others. In turn, lindane caused an increase in SOD activity in thymic cells [59,60].

In the case of catalase, a slight decrease in enzyme activity was observed with increasing concentrations of the nitroxides used, but the results were not statistically significant. A similar tendency was observed among piperidine nitroxides [47]. The observed tendency to decrease catalase activity after the treatment of RBCs with nitroxides may have been caused by a decrease in NADPH concentration, which may partially participate in their reduction. It was shown that a decrease in NADPH concentration in RBCs with glucose-6-phosphate dehydrogenase (G6PD) deficiency resulted in a decrease in catalase activity. It was found that the addition of NADPH to normal or G6PD-deficient hemolysates fully restored the activity of catalase in the decomposition of hydrogen peroxide [61]. Interestingly, LDH can catalyze one-electron oxidation of NADH to produce H_2_O_2_, which leads to a decrease in NADH concentration [62,63]. In cells, superoxide should initiate LDH to produce H_2_O_2_ similarly to superoxide in an aqueous solution [64].

The next enzyme tested was lactate dehydrogenase. The results clearly showed that none of the five-membered nitroxides influenced the activity of this enzyme. Our previous studies showed that only Tempol significantly reduced LDH activity. We also investigated the possibility of oxidation of hemoglobin to methemoglobin in RBCs treated with nitroxides. Interestingly, only pyrroline nitroxides at the highest concentrations led to the oxidation of hemoglobin to MetHb (Figure 5). Among the six-membered nitroxides, Tempo had the highest activity in Hb oxidation, and Tempamine had the lowest activity. Oxidation of Hb to MetHb was also observed in another work. Ferricyanide, which does not penetrate the plasma membranes of RBCs, added to the blood cell suspension fully inhibited Hb oxidation [48]. It was also shown that indolinic and quinolinic nitroxides (1,2-dihydro-2-ethyl-2-phenyI-3*H*-indole-3-phenylimino-1-oxyl and 1,2-dihydro-2,2-diphenyl4-ethoxyquinoline-1-oxyl, respectively) initiated hemolysis of trout RBCs. Moreover, this initiated the oxidation of hemoglobin to the MetHb form [65]. Finally, we determined the effect of nitroxides on the initiation of oxidative stress in RBCs. We showed that all nitroxides used did not cause lipid peroxidation or protein oxidation.

Summarizing our work related to the effect of nitroxides on RBCs, it can be noted that all nitroxides modulate the antioxidant system of red blood cells, starting with low-molecular-weight antioxidants such as glutathione and ascorbic acid and ending with the enzymatic system. All seven nitroxides induced an increase in SOD activity, although the smallest effect was observed for Carboxy-Pyrrolide and the largest for Pyrrolide. All nitroxides did not affect catalase activity. On the other hand, piperidine nitroxides and carboxy-Pyrrolin reduced GPx activity. In the case of GR, only Tempamine and Pyrrolin significantly reduced the activity of this enzyme. However, all nitroxides reduced GST activity, starting from a concentration of 0.2 mmol. A decrease in the concentration of antioxidants and a decrease in enzyme activity (except SOD) indicate their negative impact on red blood cells. To this, we can also add the oxidation of hemoglobin by piperidine nitroxides and Pyrrolin and Carboxy-Pyrrolin in the highest concentrations. However, a positive aspect of their action is the induction of the level of thiol groups in plasma membrane proteins and hemolysate.

Interestingly, the half-life of piperidine nitroxides in erythrocytes is approximately 1 h. For comparison, the half-life of five-membered nitroxides is at least five to six times longer. This indicates that the structure heterocyclic ring is of key importance. Looking at the spatial structure of nitroxides, the nitroxide group in piperidine nitroxides is more accessible to agents that can react with it.

## 4. Materials and Methods

### 4.1. Chemicals

The nitroxides Tempo (2,2,6,6-tetramethylpiperidine-1-oxyl, 2,2,6,6-tetramethylpiperidin-1-oxide UPAC name), Tempol (4-hydroxy-2,2,6,6-tetramethylpiperidine-1-oxyl, (4-hydroxy-2,2,6,6-tetramethylpiperidin-1-yl)oxyl UPAC name), Tempamine (4-amino-2,2,6,6-tetramethylpiperidine-1-oxyl, 2,2,6,6-tetramethyl-4-piperidinamine 1-oxide UPAC name), Carbamoyl-Pyrrolin (3-carbamoyl-2,2,5,5-tetramethyl-3-pyrroline-1-oxyl, (3-carbamoyl-2,2,5,5-tetramethyl-2,5-dihydro-1*H*-pyrrol-1-yl)oxidanyl UPAC name), Carboxy-Pyrrolin (3-carboxy-2,2,5,5-tetramethyl-3-pyrroline-1-oxyl, (3-carboxy-2,2,5,5-tetramethyl-2,5-dihydro-1*H*-pyrrol-1-yl)oxidanyl UPAC name), Pyrrolid (3-carbamoyl-2,2,5,5-tetramethyl-3-pyrrolidine-1-oxyl, (3-carbamoyl-2,2,5,5-tetramethyl-1-pyrrolidinyl)oxidanyl UPAC name), and Carboxy-Pyrrolid (3-carboxy-2,2,5,5-tetramethyl-3-pyrrolidine-1-oxyl, (3-carboxy-2,2,5,5-tetramethyl-2,5-dihydro-1*H*-pyrrol-1-yl)oxidanyl UPAC name) came from MERCK (Rahway, NJ, USA).

### 4.2. Material

The research was conducted using RBCs obtained from healthy donors. Blood was collected into 3.8% sodium citrate in a ratio of 9:1. The blood was then centrifuged for 10 min at 3000 rpm to remove plasma and leukocytes. The isolated RBCs were washed three times with PBS (10 mM phosphate-buffered saline, pH 7.4) that was cooled to 4 °C. After that, a suspension was prepared with a hematocrit of 10% in PBS, and it was incubated for 1 h at room temperature (20–22 °C) with the appropriate nitroxides. The samples contained different concentrations of nitroxides: 0.2 mM, 0.5 mM, 1 mM, and 2 mM. A control sample was included in each repetition, which consisted of RBCs incubated without nitroxide. After incubation, the samples were centrifuged and the RBCs were washed three times with 10 times the volume of cold PBS.

### 4.3. Hemolysate and RBC Membrane Preparation

The RBC plasma membranes were extracted from RBCs using the method described by Dodge et al. [66]. The concentration of plasma membrane proteins was measured using Folin–Ciocalteu reagent. Spectrophotometry was used to measure the concentrations of the proteins, with an absorption maximum of 750 nm [67].

To prepare the hemolysate, RBCs were mixed with cold water in a ratio of 1:1.5. The samples were then centrifuged to remove RBC membranes. The hemoglobin (Hb) concentration in the hemolysate was determined spectrophotometrically as cyanmethemoglobin using Drabkin’s reagent at an absorption maximum of 540 nm [68]. All spectrophotometric measurements were made at room temperature (20–22 °C).

### 4.4. Determination of Total Glutathione Concentration (GSH + GSSG) in Hemolysate

The method for determining total glutathione concentration is the reduction (via NADPH) of the total glutathione pool available to the cell [69]. The measure of the total concentration of glutathione in the cell is the rate of increase in absorbance at a wavelength of λ = 412 nm. By measuring the increase in absorbance over time at this wavelength, the concentration of glutathione in the tested samples was determined based on a standard curve for GSSG solutions of known concentrations. The results are presented as percentage changes relative to the control.

### 4.5. Determination of Ascorbate Concentration in Hemolysate

To determine the concentration of ascorbate in RBCs, a modified method was developed using bathophenanthroline (4,7-diphenyl-1,10-phenanthroline), which forms a colored complex with Fe(II) ions [70]. Excess Fe^3+^ ions were added to the reaction mixture, which was reduced to Fe^2+^ in the presence of ascorbate. The absorbance of the chloroform layer was determined at a wavelength of 525 nm. The ascorbate concentration was determined based on a standard curve prepared for known ascorbate concentrations and presented as percentage changes relative to the control.

### 4.6. Determination of the Concentration of Thiol Groups in RBC Membranes and Hemolysate Proteins

The quantity of -SH groups in RBC membrane proteins was measured using the Ellman method [71] via spectrophotometry. Ellman’s reagent (DTNB; 5,5′-dithiobis (2-nitrobenzoic acid)) reacts with free thiol groups and produces optically active 2-nitro-5-thiobenzoate (NTB) that can be detected at 412 nm.

The level of thiol groups in the hemolysate was measured using 4,4′-dithiodipyridine [72], which reacts with thiol groups of proteins and creates 2-thiopyridone. The absorption maximum of 2-thiopyridone occurs at 324 nm.

Calibration curves were created for both methods using different concentrations of reduced glutathione. Based on these curves, the concentration of thiol groups was calculated and expressed in nmol/mg protein and nmol/mg Hb.

### 4.7. Superoxide Dismutase Activity Assay

Superoxide dismutase (SOD) activity in the hemolysate was determined by the adrenaline method in carbonate buffer with a pH of 10.2 [73]. The concentration of the formed adrenochrome was measured spectrophotometrically at a wavelength of λ = 480 nm. The absorbance was measured depending on time. The volume of hemolysate that inhibited the auto-oxidation of adrenaline by 50%, which is one unit of enzyme activity, was determined and then converted into the amount of Hb in this volume. SOD activity was expressed as U/(mg Hb/min).

### 4.8. Determination of Catalase Activity

Catalase activity was determined according to the method described by Aebi [74], which involves monitoring the rate of hydrogen peroxide decomposition by the tested enzyme. The decomposition rate of hydrogen peroxide was determined spectrophotometrically at a wavelength of λ = 240 nm. A decrease in the absorbance of hydrogen peroxide equal to 0.036 absorbance unit/min equals the amount of enzyme that decomposes 1 mM of H_2_O_2_, corresponding to one unit of activity. Catalase activity was expressed in U/(mg Hb/min).

### 4.9. Lactate Dehydrogenase Activity Assay

Lactate dehydrogenase (LDH) activity in the hemolysate was determined using the method described by Wroblewski and Ladue [75]. Lactate dehydrogenase catalyzes the reaction of converting pyruvate to lactate, in which the proton donor is the reduced NADH. The principle of this method is based on determining the rate of oxidation of NADH to NAD+. The rate of NADH disappearance was determined spectrophotometrically by measuring the decrease in absorbance at a wavelength of λ = 340 nm over 0.5. The obtained results were expressed as U/(mg Hb/min).

### 4.10. Determination of the Degree of Hemoglobin Oxidation (% MetHb)

The percentage of methemoglobin in the total hemoglobin pool was determined spectrophotometrically. The absorbance of the hemolysate was measured at two wavelengths: λ = 630 and λ = 700 nm; then, potassium ferricyanide was added to the samples, thus converting all the hemoglobin into the MetHb form, and the absorbance was measured again at the same wavelengths using the following formula:

$$\%MetHb=\frac{A630-A700}{A630*-A700*}\cdot 100\%$$

where A_630_ and A_700_ represent the absorbances of hemolysate, and A_630_∗ and A_700_∗ represent the absorbances of hemolysate with potassium ferricyanide at 630 and 700 nm, respectively.

The percentage of MetHb was calculated.

### 4.11. Determination of the Thiobarbituric Acid-Reactive Substances

The level of thiobarbituric acid-reactive (TBA-reactive) compounds was measured in the hemolysate using the method outlined in a study by Stocks and Dormandy [76]. When the end products of lipid oxidation react with thiobarbituric acid at a low pH (TBARSs), optically active products are formed that can be measured at 535 nm. Changes in the concentration of compounds reacting with TBA were compared to the control and expressed as percentages.

### 4.12. Determination of the Concentration of Carbonyl Groups

The degree of oxidative damage to proteins was determined according to the method described by Levine et al. [77]. The method involves reacting 2,4-dinitrophenylhydrazine (DNPH) with carbonyl groups of oxidized proteins. The concentration of carbonyl groups was determined spectrophotometrically at a wavelength of λ = 370 nm. The values were then calculated using the millimolar absorption coefficient of 21.01 mmol^−1^ × cm^−1^, and the final results were expressed in nmol/mg Hb.

### 4.13. Statistical Analysis of the Obtained Results

To analyze the data accurately, certain statistical tests were performed. Firstly, the distribution of the tested parameters was checked for normality using the Shapiro–Wilk test. Next, the homogeneity of variances was checked using Levene’s test. Based on the results of these tests, a one-way ANOVA was conducted using a post hoc Tukey multiple comparisons test. A *p*-value of less than 0.05 indicated statistical significance. The software used for conducting the statistical analysis was Statistica v. 13.3 (StatSoft Polska, Krakow, Poland).

## 5. Conclusions

The decrease in the glutathione concentration and in the activity of glutathione-dependent enzymes, such as glutathione peroxidase, glutathione transferase, and glutathione reductase, demonstrated in our previous works [47,49], as well as the decrease in the concentration of ascorbic acid and the increase in the content of methemoglobin and in the activity of superoxide dismutase, reveal the pro-oxidant properties of nitroxides in human red blood cells.

## Data Availability

Data are contained within the article.

## Abbreviations

AA	ascorbic acid
Carboxy-Pyrrolin	3-carboxy-2,2,5,5-tetramethylpyrroline-1-oxyl
Carboxy-Pyrrolid	3-carboxy-2,2,5,5-tetramethylpyrrolidine-1-oxyl
CAT	catalase
DHA	dehydroascorbate
GSH	glutathione
GSSG	oxidized glutathione
GPx	glutathione peroxidase
GRx	glutathione reductase
GT	glutathione transferase
Grx	glutaredoxin
G6PD	glucose-6-phosphate dehydrogenase
Hb	hemoglobin
LDH	lactate dehydrogenase
Mb	myoglobin
MetHb	methemoglobin
NAD^+^	nicotinamide adenine dinucleotide oxidized form
NADH	nicotinamide adenine dinucleotide reduced form
RBCs	red blood cells (erythrocytes)
TBARSs	thiobarbituric acid-reactive substances
Tempo	2,2,6,6-tetramethylpiperidine-1-oxyl
Tempol	4- hydroxy-2,2,6,6-tetramethylpiperidine-1-oxyl
Tempamine	4-amine-2,2,6,6-tetramethylpiperidine-1-oxyl
Pyrrolin	3-carbamoyl-2,2,5,5-tetramethylpyrroline-1-oxyl
Pyrrolid	3-carbamoyl-2,2,5,5-tetramethylpyrrolidine-1-oxyl
SOD	superoxide dismutase
Trx	thioredoxin
